# Mortality, Morbidity and Health-Seeking Behaviour during the Ebola Epidemic 2014–2015 in Monrovia Results from a Mobile Phone Survey

**DOI:** 10.1371/journal.pntd.0004899

**Published:** 2016-08-23

**Authors:** Anna Kuehne, Emily Lynch, Esaie Marshall, Amanda Tiffany, Ian Alley, Luke Bawo, Moses Massaquoi, Claudia Lodesani, Philippe Le Vaillant, Klaudia Porten, Etienne Gignoux

**Affiliations:** 1 Postgraduate Training for Applied Epidemiology, Robert Koch Institute, Berlin, Germany affiliated to the European Programme for Intervention Epidemiology Training, ECDC, Stockholm, Sweden; 2 Epicentre, Paris, France; 3 Epicentre, Geneva, Switzerland; 4 Ministry of Health, Monrovia, Liberia; 5 Médecins sans Frontières, Paris, France; Common Heritage Foundation, NIGERIA

## Abstract

Between March 2014 and July 2015 at least 10,500 Ebola cases including more than 4,800 deaths occurred in Liberia, the majority in Monrovia. However, official numbers may have underestimated the size of the outbreak. Closure of health facilities and mistrust in existing structures may have additionally impacted on all-cause morbidity and mortality. To quantify mortality and morbidity and describe health-seeking behaviour in Monrovia, Médecins sans Frontières (MSF) conducted a mobile phone survey from December 2014 to March 2015. We drew a random sample of households in Monrovia and conducted structured mobile phone interviews, covering morbidity, mortality and health-seeking behaviour from 14 May 2014 until the day of the survey. We defined an Ebola-related death as any death meeting the Liberian Ebola case definition. We calculated all-cause and Ebola-specific mortality rates. The sample consisted of 6,813 household members in 905 households. We estimated a crude mortality rate (CMR) of 0.33/10,000 persons/day (95%CI:0.25–0.43) and an Ebola-specific mortality rate of 0.06/10,000 persons/day (95%-CI:0.03–0.11). During the recall period, 17 Ebola cases were reported including those who died. In the 30 days prior to the survey 277 household members were reported sick; malaria accounted for 54% (150/277). Of the sick household members, 43% (122/276) did not visit any health care facility. The mobile phone-based survey was found to be a feasible and acceptable alternative method when data collection in the community is impossible. CMR was estimated well below the emergency threshold of 1/10,000 persons/day. Non-Ebola-related mortality in Monrovia was not higher than previous national estimates of mortality for Liberia. However, excess mortality directly resulting from Ebola did occur in the population. Importantly, the small proportion of sick household members presenting to official health facilities when sick might pose a challenge for future outbreak detection and mitigation. Substantial reported health-seeking behaviour outside of health facilities may also suggest the need for adapted health messaging and improved access to health care.

## Author Summary

During the Ebola outbreak in 2014/2015 more than 4,800 people died of Ebola in Liberia. Health care providers in the field have assumed that closure of health facilities and mistrust in existing structures resulted not only substantial additional deaths from Ebola but also impacted on death rate of other diseases and on the way people tried to seek health care. We conducted a mobile phone survey in Monrovia to identify deaths and diseases a household had faced since the beginning of the Ebola outbreak and the kind of health care they sought. We estimated that the non-Ebola-related death rate in Monrovia was not higher than previous national estimates for Liberia. However, additional deaths occurred in the population of Monrovia directly resulting from Ebola. Of the household members that were sick of any disease during the survey period, 43% did not visit any health care facility. The high proportion among the sick household members that sought health care in pharmacies or drug stores or by health care workers among their peers but outside health facilities emphasizes the importance of ensuring access to non-Ebola-related health care as outbreak control is challenged by sick community members staying undiagnosed and untreated.

## Introduction

Between March 2014 and July 2015, more than 10,500 Ebola virus disease (EVD) cases, including over 4,800 deaths, occurred in Liberia; the majority of these cases was identified in Montserrado County, where the capital city of Liberia, Monrovia, is located [1].

However, official reported numbers of EVD cases might underestimate the size of the outbreak for several reasons: i) during the intense phase of the outbreak in August 2014, Ebola treatment units (ETUs) were overwhelmed with patients, some of whom were turned away uncounted [2]; ii) the continued identification of cases that had not been registered as contacts of known EVD cases beforehand indicates that contact tracing remained incomplete throughout the outbreak and cases are likely to have been missed [3] and iii) communities hesitated to send sick members to ETUs [2,4]. A capture-recapture study conducted in August 2014 suggested that actual case numbers could be three times higher than the number of notified EVD cases [5].

Health care providers in the field have assumed that closure of health facilities and mistrust in existing structures resulted not only in EVD-related excess morbidity and mortality but also had an impact on non-EVD morbidity and mortality: In Monrovia—similar to what has been described in other affected countries and cities [6]–the capacity of health care facilities was greatly reduced in August 2014 as compared to prior to the epidemic [7–10]. Even in health facilities that continued to provide health care in Monrovia, the number of consultations was reduced by at least 40% compared to previous years [7]. In addition, the health care system in Liberia lost at least 178 health care workers to EVD from the beginning of the outbreak until the end of 2014 [11]. Fear of EVD and mistrust in existing health care structures led to underutilization of services [7,8,12].

In 2013, crude mortality rates (CMR) in Liberia were reported between 0.22/10,000 persons/day [13], 0.25/10,000 persons/day [14] and 0.27/10,000 persons/day [15].

To quantify mortality and morbidity and to describe health-seeking behaviour in Monrovia during the EVD outbreak, Médecins Sans Frontières (MSF) conducted a mobile phone survey in Monrovia. The specific objectives were to a) retrospectively estimate the all-cause-mortality rate (non-EVD and EVD) during the recall period, b) retrospectively estimate disease-specific attack rates for non-EVD and EVD during the 30 days preceding the survey, c) retrospectively estimate the EVD attack rate during the recall period and d) describe health-seeking behaviour.

## Methods

### Study design, setting and period

The study was conducted in Monrovia, Liberia, from December 2014 to March 2015. Classical survey designs, such as household visits with face-to-face interviews, were determined unfeasible due to the risk of EVD transmission given the contact and movement requirements to implement a community household survey. To minimize these risks we chose instead to conduct a mobile phone based survey.

The survey covered a recall period from the 14^th^ May 2014 (National Unification Day in Liberia) to the day of the survey. The study population included the whole population of greater Monrovia. Greater Monrovia is located at the northern portion of the Liberian coast at the mouth of the Mesurado River and extends across a series peninsulas and wetlands. Greater Monrovia consists of a population of about 1 million inhabitants (MSF operational data) and is the most urbanized area in Liberia, its capital and main port. Telecommunication using landlines is almost absent in most parts of Monrovia. The sampling frame therefore included households where at least one person in the household owned a Subscriber Identity Module (SIM) card from a selected large mobile phone network provider in Monrovia and had been connected to the network in Monrovia at some point in the 30 days prior to the date of the survey. The selected network provider had, according to the company, coverage of more than half of the population of Monrovia with customers in all age-groups that represented diverse socio-demographic strata.

### Sample size

The calculation of the sample size is based on the crude mortality rate and the expected EVD attack rate. For an expected crude mortality rate of 0.5/10,000/day based on doubling of the national baseline mortality [13–15], a precision of 0.15 and a recall period of 255 days (from 15 May 2014 to mid-survey date, 24 January 2015), a total of 5,986 individuals in 1,197 households were needed in the sample, assuming an average household size of five household members. Assuming that real case numbers were threefold higher than those notified [5], and thus an expected attack rate of 0.51% for EVD during the whole study period (based on notified cases in the Liberian EVD patient database up to 9 November 2014 according to WHO data packages) and a precision of 0.15%, 8,660 individuals in 1,732 household were needed. Non-response was estimated at 50% as suggested by the telephone network provider, based on their usual response rates in previous telephone surveys. Therefore, 3,500 households were included in the sample.

### Sampling procedures

The network provider drew a simple random sample of telephone numbers. The network provider sent a text message to the selected customers informing them that they had been randomly selected to participate in an MSF survey. Participants sent a text message if they agreed to have their phone number forwarded to MSF and received one US Dollar of free airtime after providing this consent (regardless of whether they eventually completed the survey when contacted by the surveyors). Trained MSF surveyors made a maximum of three attempts to contact each telephone number. Respondents were only included in the survey if they were over 18 years of age, lived in greater Monrovia and consented to participation.

### Definitions

Any sickness or death was counted as EVD-related if it fulfilled the Ebola case definition of the Liberian Ministry of Health (MOH) for suspect, probable or confirmed cases [16]. A household was defined as a group of people living under the same roof and sharing the same meal at least 3 times a week regardless of family ties.

### Data collection

We trained 15 surveyors for three days to collect data using standardized questionnaires on tablets. Surveyors entered data directly into an Open Data Kit (ODK) form on the tablet during the telephone interview. At the end of each day the lead epidemiologist reviewed any recorded deaths and EVD cases together with the surveyors to ensure data quality.

Each respondent was asked to answer the questions on behalf of the entire household. For each household, the number of members of the household was recorded. Respondents were asked about any sick person in the household within the past 30 days. For each EVD case according to the case definition, information about symptoms, history of contact, isolation of the cases and burial circumstances (where applicable) was obtained. We also inquired about non-EVD deaths among household members during the recall period, along with cause of death according to the respondent and the circumstances of burial.

### Data analysis

Reported EVD cases were compared to the case definition of the Liberian Ministry of Health.

We calculated disease specific attack rates in the past 30 days–and for EVD for the whole recall period–using the sampled population at the beginning of the recall period as the denominator.

Crude mortality rate (CMR) and EVD-related-mortality-rate per 10,000 population per day were calculated using the recall period as the denominator. Household members who left, arrived or were born during the recall period were considered as having lived in the household half of the recall period. For deceased members the exact time under observation (14 May 2014 to date of death) was used as a denominator. Statistical analysis was conducted in STATA version 13 (Stata corporation, Texas, USA).

### Ethical considerations

The procedures conducted were in accordance with the ethical standards of the Helsinki Declaration. Additionally, the National Research Ethics Board of the Ministry of Health of Liberia granted ethical approval and authorization to conduct this survey.

An information sheet was read and explained to each respondent at the beginning of the interview. Participation was voluntary. For each respondent, oral informed consent was requested and this response was documented. No personal identifiable information was collected. Telephone numbers were kept confidential and stored securely.

## Results

### Response and household size

768 (22%) persons of 3,500 that were contacted by the network provider from 19 December to 2 January 2015 responded to the text message and agreed to be contacted for the survey, 446 were reached by the surveyors and fulfilled the required eligibility criteria (overall response rate 13%; 446/3,500) (Table 1). They provided information for 3,363 household members, indicating an average household size of 7.5 persons per household.

**Table 1:** Response in two rounds of sampling; mortality, morbidity and health-seeking behaviour survey during the Ebola epidemic in Monrovia, Liberia, May 2014-March 2015.

Response		Round 1		Round 2		Total	
		19Dec14-02Jan15		29Jan15-07Mar15		19Dec14-07Mar15	
		N = 3,500		N = 7,000		N = 10,500	
		n	%	n	%	n	%
Customers responding to text message		768	22%	781	11%	1549	15%
Customers reached in three tries by cell phone		630	18%	684	9.8%	1314	13%
	Respondent did not consent	49	1,4%	38	0,5%	87	0,8%
	Respondents under 18 years	41	1,2%	45	0,6%	86	0,8%
	Respondent did not live in greater Monrovia	94	2,7%	142	2,0%	236	2,2%
Customers included into the survey		446	13%	459	6.6%	905	8.6%

As a result of the response outcome through early January 2015, the sample size was recalculated, adjusting the household size estimate (5 → 7.5) and the expected non-response rate (50% → 90%). A second round of sampling was launched in order to reach the required numbers of respondents, with an additional 7,000 phone numbers contacted by text (total contacted, 10,500).

Of the 7,000 texts messages sent to a new random sample of mobile phone owners between 29 January and 7 March 2015, 781 customers replied with agreement to participate (11%; 781/7,000) (Table 1). 459 households were eligible for inclusion (Table 1); they represented 3,450 household members (average household size 7.5).

After both rounds of sampling, the survey included a total of 905 households. The total number of days in the recall period ranged from 215 days (first interview) to 293 days (last interview).

### Sample characteristics

At the beginning of the survey period, 6,813 household members were included in the sample (Table 2). The crude birth rate was 29.1 births per 1,000 persons per year.

**Table 2:** Changes in household composition among respondents; mortality, morbidity and health-seeking behaviour survey during the Ebola epidemic in Monrovia, Liberia, May 2014-March 2015.

Household members	Frequency	Percent
Household members on 14 May 2014	6813	100%
Household members that left during the recall period	481	7.1%
Household members that arrived during the recall period	291	4.3%
Births during the recall period	133	2.0%
Non-Ebola deaths during the recall period	45	0.7%
Ebola deaths during the recall period	10	0.1%
Household members at the day of the survey	6701	98.4%

The median age of the 905 respondents was 29 years (interquartile range: 23–36). Respondents came from all parts of Monrovia; between 31 and 146 persons/10,000 population per neighbourhood participated in the survey (Table 3). The proportion of households included was lower for precarious neighbourhoods and higher in more affluent neighbourhoods.

**Table 3:** Number of households And household members included in the survey by neighbourhood of residence; mortality, morbidity and health-seeking behaviour survey during the Ebola epidemic in Monrovia, Liberia, May 2014-March 2015.

neighbourhood in Monrovia	population size (MSF operational data)	Households in the sample	Household members in the sample	Median house-hold size	Persons included per 10,000 population
Barnesville	36,014	64	462	7.2	128
Caldwell	27,754	48	405	8.4	146
Central Monrovia	85,819	63	421	6.7	49
Clara Town	56,446	27	193	7.1	34
Congo Town	n.a.	42	273	6.5	n.a.*
Gardnersville	81,590	73	558	7.6	68
Lapkazee	40,753	16	127	7.9	31
Logan Town	56,350	36	337	9.4	60
New Georgia	58,958	40	259	6.5	44
New Kru Town	75,191	34	357	10.5	47
Old Road	49,012	43	282	6.6	58
Paynesville	330,066	344	2,569	7.5	78
Sinkor	42,041	65	465	7.2	111
Westpoint	30,830	10	105	10.5	34
Total	939,994	905	6813	7.5	72

n.a. = not available

### Mortality rates for all causes of death (non-EVD and EVD) during the recall period

Overall 55 deaths occurred during the recall period, indicating a crude mortality rate of 0.33/10,000 (95%-CI: 0.25–0.43) persons per day. Fifty-five deaths occurred in 47 households, indicating that over the entire recall period, 5.2% of households experienced at least one death. Six (0.7%) households experienced more than one death.

Forty-five (82%) of 55 deaths did not meet the EVD case definition, indicating a non-EVD mortality rate of 0.27/10,000 (95%-CI: 0.20–0.36) persons per day.

Deaths due to a cause other than EVD were chronic diseases (n = 20), injuries (n = 6), birth- or pregnancy-associated deaths (n = 3), “African sign” (n = 3), ulcers (n = 2), “old age” (n = 1), stomach pain (n = 1), food poisoning (n = 1), “no good care” (n = 1) and jaundice (n = 1). For six deaths the reason was unknown.

EVD attributable deaths were 10 of 55 (18%; 95%-CI: 9–31%), indicating an EVD specific mortality rate of 0.06/10,000 (95%-CI:0.03–0.11) persons per day. Individuals that died of EVD had a lower median age than those dying of non-EVD causes (median age 32 and 36 years, respectively; interquartile range 25–33 and 21–56, respectively) and were more frequently reported to have died in an ETU (Table 4).

**Table 4:** Characteristics of deceased cases (n = 55); mortality, morbidity and health-seeking behaviour survey during the Ebola epidemic in Monrovia, Liberia, May 2014-March 2015.

	EVD* related death (N = 10)		Death from other causes (N = 45)	
	n	%	n	%
Sex male	4	40%	23	51%
Places of death				
Public or private Health facility	2	20%	15	33%
Ebola treatment units	6	60%	5	11%
Home	2	20%	23	51%
Another location	-	-	2	4%
Body disposal by specialized burial team	9	90%	5	11%

Six (11%) of 55 deaths were of children under 5 years of age; their causes were reported as EVD (n = 1), asphyxia at birth (n = 1), “African sign” (n = 1), “no good care” (n = 1) and ulcers (n = 2).

### Morbidity—Disease specific attack rates for non-EVD and EVD during the 30 days preceding the survey

277 (4%) of 6,813 household members were reported to have been sick in the 30 days prior to the survey. 19 of those sick reported suffering from more than one disease, resulting in 277 sick persons with 295 disease episodes. The most frequently reported diseases were malaria (2.2% of all household members), acute respiratory infections (ARI) (0.4%) and chronic diseases (0.3%) (Table 5). One EVD case was reported during the 30 days preceding the survey.

**Table 5:** Respondent-reported morbidity; mortality, morbidity and health-seeking behaviour survey during the Ebola epidemic in Monrovia, Liberia, May 2014-March 2015.

Self-diagnosis	Frequency of disease within the past 30 days	Attack rate among household members (N = 6813)	Percentage of sick people (N = 277)
Malaria	150	2,2%	54,2%
Acute respiratory infection	28	0,4%	10,1%
Chronic diseases	21	0,3%	7,6%
Headache	15	0,2%	5,4%
Typhoid	11	0,2%	4,0%
Trauma	11	0,2%	4,0%
Diarrhoea	6	0,1%	2,2%
Stomach pain	4	0,1%	1,4%
Constipation	4	0,1%	1,4%
Arthritis	4	0,1%	1,4%
Worms	2	0,0%	0,7%
Fever	2	0,0%	0,7%
Pregnancy-related illness	2	0,0%	0,7%
Tooth decay	2	0,0%	0,7%
Ebola	1	0,0%	0,4%
Allergy	1	0,0%	0,4%
Back pain	1	0,0%	0,4%
Body pain	1	0,0%	0,4%
Skin rash	1	0,0%	0,4%
Fatigue	1	0,0%	0,4%
Food poison	1	0,0%	0,4%
Hernia	1	0,0%	0,4%
Throat pain	1	0,0%	0,4%
Swollen lower extremity	1	0,0%	0,4%
Urinary tract infection	1	0,0%	0,4%
Jaundice	1	0,0%	0,4%
Unknown	22	0,3%	7,9%
Total	295	4,3%	106,5%

10 (4%) of the 277 household members that had been sick in the 30 days prior to the survey were children under five years of age, 70% (7/10) of these children were reported to have had experienced an episode of malaria.

### Morbidity—Attack rate of EVD during the recall period

19 EVD cases were reported to have occurred between 14 May 2014 and the date of interview. However, three of the reported EVD cases did not meet case definition. Of the remaining 16 cases, four met the case definition for a suspect case, one for a probable case and eleven for a confirmed case. One additional death reported as a non-EVD death met the case definition for a suspect case. The overall attack rate of reported cases meeting the EVD case definition (n = 17) among all household members was 0.25% (95%-CI: 0.15–0.40). Of 17 EVD cases, 10 were reported to have died (case fatality rate: 59%).

**Fig 1:**
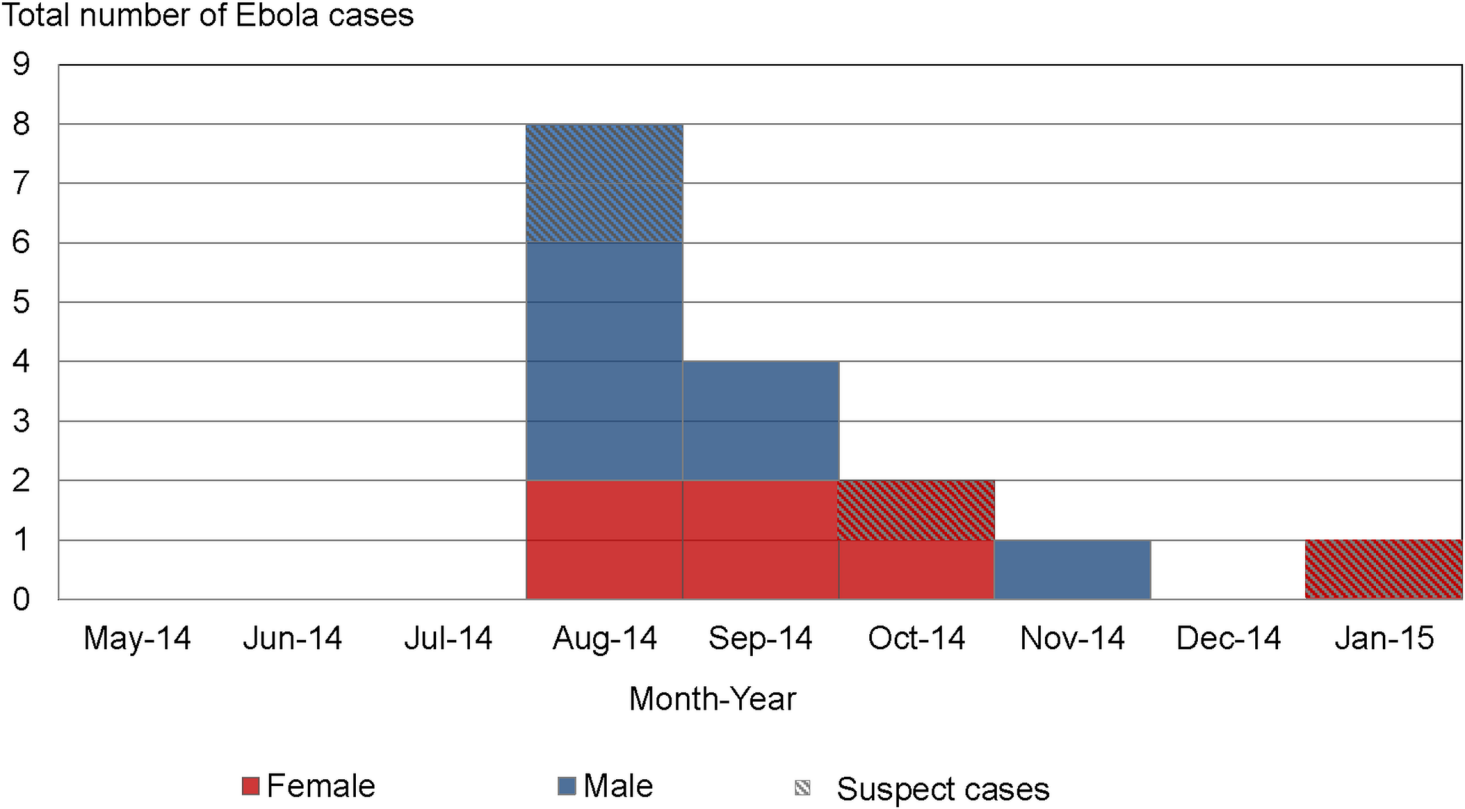
Number of Ebola cases by month of disease onset (n = 16); mortality, morbidity and health-seeking behaviour survey during the Ebola epidemic in Monrovia, Liberia, May 2014-March 2015.

Median age of the reported EVD cases was 29 years, 53% of cases were male. 76% were reported to have been hospitalised in ETUs. Date of symptom onset was available for 16 cases, eight of which had symptom onset in August, after which the number of cases declined (Fig 1).

### Health-seeking behaviour

Nine (3%) of the 276 individuals, who had been sick during the 30 days prior to the survey with non-EVD diseases, did not seek any health care. Forty-three (16%) of the 276 sick people accessed health care in a government-run health facility. 112 (41%) accessed health care in private health facilities. 54 (20%) found treatment either in pharmacies or drug stores and 50 (18%) asked a health care worker (HCW) to take care of them at home, 6 (2%) visited a traditional healer, one sought healthcare at the church and one elsewhere.

Of the 150 reported cases of malaria, 35% (52/150) did not seek care at neither a public nor a private health facility; an additional 16% (24/150) were reported to have been treated at home by a health care worker.

## Discussion

The estimated CMR and its confidence intervals were considerably below the emergency threshold of 1/10,000 persons per day [17]. The non-EVD-related mortality was similar to national estimates of the mortality rate prior to the outbreak for Liberia [13–15], suggesting that there was no increase of non-EVD-related mortality during the EVD outbreak to the extent initially expected by actors in the field. A similar observation was made in a mortality survey conducted in Sierra Leone’s capital Freetown that indicated no increase in non-EVD mortality during the EVD outbreak [18]. In fact the strict hygiene measure implemented to prevent Ebola transmission, such as limited movements, no touch policy and rigorous hand-washing might have positively impacted on non-EVD-related mortality and morbidity.

However, it is possible that the mortality rate in Monrovia prior to the Ebola epidemic was lower than the countrywide estimates, as access to health care was better in Monrovia compared with other parts of the country [10,19]. Furthermore our mortality estimate might be underestimated; mortality among children under five years of age accounted for only 11% of reported deaths and seems to be insufficiently represented as mortality amongst children under five is usually higher than amongst adults [4,17]. Additionally, no deaths due to fever or malaria were reported, suggesting further underestimation in the sample. As respondents from more affluent neighbourhoods were overrepresented in the sample, malaria episodes might have been more often treated and resolved, resulting in lower than average numbers of malaria-related deaths.

On the other hand, malaria mortality might have in fact been low in Monrovia during part of the recall period due to a mass drug administration (MDA) of malaria chemoprevention carried out in October to December 2014 by MSF in the West part of Monrovia with a population of approximately 550,000 [23].

EVD contributed 0.06 deaths per 10,000 persons per day and was the cause of death for at least 18% of all-cause-mortality observed in the survey. In the sample an additional two deaths were reported to have happened in an ETU, but as no information on symptoms or laboratory tests was available, they were not counted as EVD deaths. Our estimates suggest that in Monrovia during the recall period, the CMR increased by 22% due to deaths directly associated with EVD. Extrapolating the EVD mortality rate observed in the study to the population of Monrovia (1,144,806 individuals [20]) indicates an estimate between 1,254 and 4,596 EVD deaths in one year in Monrovia, which is in keeping with the 2,300 EVD deaths notified to MOHSW in Monrovia up to March 2015 [21]. Thus, underestimation of EVD case notification data in Monrovia does not seem to be as high as previously estimated [5].

More than half of the reported morbidity was due to malaria, the disease reported to be one of the main reasons for outpatient consultations in Liberia [22]. The estimated attack rate for malaria was in accordance with those found in other surveys at the end of 2014 in Monrovia [23]. EVD accounted for 0.4% of morbidity within the month prior to the survey. However, EVD case numbers were sharply decreasing after August 2014—in the sample and in national notification data–thus EVD probably accounted for a larger proportion of morbidity earlier in 2014.

76% of EVD cases were reported to have been isolated in an ETU, indicating some functionality and trust in EVD-related treatment facilities. On the other hand, respondents might have been reluctant to report if they were in fact not admitted.

Of the household members experiencing any illness, 43% did not seek care at a health facility, a proportion consistent with that of previous studies [24]. Low utilisation of public health facilities for illnesses other than EVD may have resulted in under-diagnosis and no treatment of diseases. However, EVD-hygiene-measures, the MDA and alternative care seeking behaviour, such as home treatment and the use of local pharmacies, may have contributed to little or no observed increase in mortality. Given the low proportion of health facility utilisation, more comprehensive and timely implementation of EVD triage training (combined with functioning and trusted telephone help lines) provided to local drug store keepers and any health care worker–even unemployed–might have been key to prevent EVD spread by home treatment.

The proportion of health care seeking at private health facilities might have been lower in less affluent parts of Monrovia that are underrepresented in this sample and might add to the underestimation of mortality.

### Feasibility of the mobile phone survey and its limitations

We have shown that a mobile phone survey can be implemented in a context where access to the population is limited–be it due to the risk of infection by Ebola, as in this case or, potentially to other contexts such as insecure conflict environments, difficulties in transportation during rainy season or after natural disasters, or difficult access caused by the risk of kidnapping.

There are several limitations introduced by using the mobile telephone methodology:

First, the dependence on a network provider for random sampling created some delay and contributed to a lack of full transparency in the process.

Second, data on population coverage of the network provider in Monrovia was unknown as were the socio-demographic characteristics of customers, leading to uncertainties regarding the representativeness of the sample for Monrovia’s whole population. Low rates of inclusion of households in precarious neighbourhoods of Monrovia suggest that the sample frame might not have been as representative of the population of greater Monrovia as implied by the provider. Thus, poverty associated diseases and deaths, including EVD, may be underestimated in this survey. Despite these limitations, mean age of the respondents and household size was similar to previously published estimates for Monrovia [23] and estimates for birth and death rates [15], morbidity [22,23] and health seeking behaviour [24] were in accordance with previous research indicating acceptable representation of Monrovia’s population. In future mobile phone surveys underrepresentation could possibly be mitigated with an adapted quota sampling strategy, if sociodemographic and -economic characteristics of the customers of the partnering company are available.

Third, the respondents’ environment can have an influence on the quality of data gathered from mobile phone surveys. Mobile phone respondents can be in situations where survey participation is not feasible or the connection not adequate. Despite these potential limitations, studies have shown that the interview mode does not influence significantly the quality of data measurement [25].

Fourth, the initial response was much lower than expected by the provider but in keeping with response in telephone surveys performed in industrialized countries [26]. The low response necessitated a new round of sampling and extended the recall period. Our experience suggests that for future mobile phone based surveys, the sample size calculations should account for a proportion of non-response as high as in industrialized countries.

As with all retrospective mortality studies, there is the potential for recall and survivor bias that may have affected the results. Additionally, stigma, fear, an environment of mistrust and the trauma of the experience may have led to under-reporting of EVD; the format of the survey–dependent on the reporting of sensitive health information to an unknown surveyor on the phone–may have exacerbated this effect.

For operational reasons–given the context and the “no-touch-policy”–we could not compare the telephone survey method with a gold standard community based survey to evaluate the specificity and sensitivity.

### Conclusion

The mobile phone based survey was found to be a feasible and acceptable alternative method when data collection in the community is impossible.

CMR was estimated below the emergency threshold of 1/10,000 persons per day. Our results suggest excess mortality in the population directly resulting from Ebola. However, non-EVD mortality did not increase to the extent originally expected.

Importantly, the small proportion of sick household members presenting to official health facilities when sick might pose a challenge for future outbreak detection and mitigation. Substantial reported health-seeking behaviour outside of health facilities may also suggest the need for adapted health messaging and improved access to health care.
